# Supplementary material for: Alterations to the broad-spectrum formin inhibitor SMIFH2 modulate potency but not specificity
Source: Sci Rep. 2022 Aug 8;12:13520. doi: 10.1038/s41598-022-17685-z (PMC9360399; doi:10.1038/s41598-022-17685-z)
Supplement: Supplementary file 1 — Supplementary Information 1. [file 41598_2022_17685_MOESM1_ESM.rtf]

Text file 1. Sequences of formin constructs.DIAPH1-FFC (Transomic BC117257, aa 549–1262)* One proline deletion from NP_005210.3, aa 558-1272     549  550  551  552  553  554  555  556  557  558  559  560  561  562  563  564  565  566  567  568  569  570  571  572  M    A    K    K    E    M    A    S    L    S    A    A    A    I    T    V    P    P    S    V    P    S    R    A    P    ATG  GCC  AAG  AAA  GAA  ATG  GCT  TCC  CTC  TCT  GCG  GCA  GCT  ATT  ACT  GTA  CCT  CCT  TCT  GTT  CCT  AGT  CGT  GCT  CCT  573  574  575  576  577  578  579  580  581  582  583  584  585  586  587  588  589  590  591  592  593  594  595  596  597  V    P    P    A    P    P    L    P    G    D    S    G    T    I    I    P    P    P    P    A    P    G    D    S    T    GTT  CCC  CCT  GCC  CCT  CCT  TTA  CCT  GGT  GAC  TCT  GGC  ACT  ATT  ATT  CCA  CCA  CCA  CCT  GCT  CCT  GGG  GAT  AGT  ACC  598  599  600  601  602  603  604  605  606  607  608  609  610  611  612  613  614  615  616  617  618  619  620  621  622  T    P    P    P    P    P    P    P    P    P    P    P    P    L    P    G    G    V    C    I    S    S    P    P    S    ACT  CCT  CCT  CCT  CCT  CCT  CCT  CCT  CCT  CCT  CCA  CCT  CCT  TTG  CCT  GGG  GGT  GTT  TGC  ATC  TCC  TCA  CCC  CCT  TCT  623  624  625  626  627  628  629  630  631  632  633  634  635  636  637  638  639  640  641  642  643  644  645  646  647  L    P    G    G    T    A    I    S    P    P    P    P    L    S    G    D    A    T    I    P    P    P    P    P    L    TTA  CCT  GGA  GGT  ACT  GCT  ATC  TCT  CCA  CCC  CCT  CCT  TTG  TCT  GGG  GAT  GCT  ACC  ATC  CCT  CCA  CCC  CCT  CCT  TTG  648  649  650  651  652  653  654  655  656  657  658  659  660  661  662  663  664  665  666  667  668  669  670  671  672  P    E    G    V    G    I    P    S    P    S    S    L    P    G    G    T    A    I    P    P    P    P    P    L    P    CCT  GAG  GGT  GTT  GGC  ATC  CCT  TCA  CCC  TCT  TCT  TTG  CCT  GGA  GGT  ACT  GCC  ATC  CCC  CCA  CCT  CCT  CCT  TTG  CCT  673  674  675  676  677  678  679  680  681  682  683  684  685  686  687  688  689  690  691  692  693  694  695  696  697  G    S    A    R    I    P    P    P    P    P    P    L    P    G    S    A    G    I    P    P    P    P    P    P    L    GGG  AGT  GCT  AGA  ATC  CCC  CCA  CCA  CCA  CCT  CCT  TTG  CCT  GGG  AGT  GCT  GGA  ATT  CCC  CCC  CCA  CCT  CCT  CCC  TTG  698  699  700  701  702  703  704  705  706  707  708  709  710  711  712  713  714  715  716  717  718  719  720  721  722  P    G    E    A    G    M    P    P    P    P    P    P    L    P    G    G    P    G    I    P    P    P    P    P    F    CCT  GGA  GAA  GCA  GGA  ATG  CCA  CCT  CCT  CCT  CCC  CCT  CTT  CCT  GGT  GGT  CCT  GGA  ATC  CCT  CCA  CCT  CCT  CCA  TTT  723  724  725  726  727  728  729  730  731  732  733  734  735  736  737  738  739  740  741  742  743  744  745  746  747  P    G    G    P    G    I    P    P    P    P    P    G    M    G    M    P    P    P    P    P    F    G    F    G    V    CCC  GGA  GGC  CCT  GGC  ATT  CCT  CCA  CCT  CCA  CCC  GGA  ATG  GGT  ATG  CCT  CCA  CCT  CCC  CCA  TTT  GGA  TTT  GGA  GTT  748  749  750  751  752  753  754  755  756  757  758  759  760  761  762  763  764  765  766  767  768  769  770  771  772  P    A    A    P    V    L    P    F    G    L    T    P    K    K    L    Y    K    P    E    V    Q    L    R    R    P    CCT  GCA  GCC  CCA  GTT  CTG  CCA  TTT  GGA  TTA  ACC  CCC  AAA  AAG  CTT  TAT  AAG  CCA  GAG  GTG  CAG  CTC  CGG  AGG  CCA  773  774  775  776  777  778  779  780  781  782  783  784  785  786  787  788  789  790  791  792  793  794  795  796  797  N    W    S    K    L    V    A    E    D    L    S    Q    D    C    F    W    T    K    V    K    E    D    R    F    E    AAC  TGG  TCC  AAG  CTT  GTG  GCT  GAG  GAC  CTC  TCC  CAG  GAC  TGC  TTC  TGG  ACA  AAG  GTG  AAG  GAG  GAC  CGC  TTT  GAG  798  799  800  801  802  803  804  805  806  807  808  809  810  811  812  813  814  815  816  817  818  819  820  821  822  N    N    E    L    F    A    K    L    T    L    T    F    S    A    Q    T    K    T    S    K    A    K    K    D    Q    AAC  AAT  GAA  CTT  TTC  GCC  AAA  CTT  ACC  CTT  ACC  TTC  TCT  GCC  CAG  ACC  AAG  ACT  TCC  AAA  GCC  AAG  AAG  GAT  CAA  823  824  825  826  827  828  829  830  831  832  833  834  835  836  837  838  839  840  841  842  843  844  845  846  847  E    G    G    E    E    K    K    S    V    Q    K    K    K    V    K    E    L    K    V    L    D    S    K    T    A    GAA  GGT  GGA  GAA  GAA  AAG  AAA  TCT  GTG  CAA  AAG  AAA  AAA  GTA  AAA  GAG  TTA  AAG  GTG  TTG  GAT  TCA  AAG  ACA  GCC  848  849  850  851  852  853  854  855  856  857  858  859  860  861  862  863  864  865  866  867  868  869  870  871  872  Q    N    L    S    I    F    L    G    S    F    R    M    P    Y    Q    E    I    K    N    V    I    L    E    V    N    CAG  AAT  CTC  TCA  ATC  TTT  TTG  GGT  TCC  TTC  CGC  ATG  CCC  TAT  CAA  GAG  ATT  AAG  AAT  GTC  ATC  CTG  GAG  GTG  AAT  873  874  875  876  877  878  879  880  881  882  883  884  885  886  887  888  889  890  891  892  893  894  895  896  897  E    A    V    L    T    E    S    M    I    Q    N    L    I    K    Q    M    P    E    P    E    Q    L    K    M    L    GAG  GCT  GTT  CTG  ACT  GAG  TCT  ATG  ATC  CAG  AAC  CTC  ATT  AAG  CAA  ATG  CCA  GAG  CCA  GAG  CAG  TTA  AAA  ATG  CTT  898  899  900  901  902  903  904  905  906  907  908  909  910  911  912  913  914  915  916  917  918  919  920  921  922  S    E    L    K    D    E    Y    D    D    L    A    E    S    E    Q    F    G    V    V    M    G    T    V    P    R    TCT  GAA  CTG  AAG  GAT  GAA  TAT  GAT  GAC  CTG  GCT  GAG  TCA  GAG  CAG  TTT  GGC  GTG  GTG  ATG  GGC  ACT  GTG  CCC  CGA  923  924  925  926  927  928  929  930  931  932  933  934  935  936  937  938  939  940  941  942  943  944  945  946  947  L    R    P    R    L    N    A    I    L    F    K    L    Q    F    S    E    Q    V    E    N    I    K    P    E    I    CTG  CGG  CCT  CGC  CTC  AAT  GCC  ATT  CTC  TTC  AAG  CTA  CAA  TTC  AGC  GAG  CAA  GTG  GAG  AAT  ATC  AAG  CCA  GAG  ATT  948  949  950  951  952  953  954  955  956  957  958  959  960  961  962  963  964  965  966  967  968  969  970  971  972  V    S    V    T    A    A    C    E    E    L    R    K    S    E    S    F    S    N    L    L    E    I    T    L    L    GTG  TCT  GTC  ACT  GCT  GCA  TGT  GAG  GAG  TTA  CGT  AAG  AGT  GAG  AGC  TTT  TCC  AAT  CTC  CTA  GAG  ATT  ACC  TTG  CTT  973  974  975  976  977  978  979  980  981  982  983  984  985  986  987  988  989  990  991  992  993  994  995  996  997  V    G    N    Y    M    N    A    G    S    R    N    A    G    A    F    G    F    N    I    S    F    L    C    K    L    GTT  GGA  AAT  TAC  ATG  AAT  GCT  GGC  TCC  AGA  AAT  GCT  GGT  GCT  TTT  GGC  TTC  AAT  ATC  AGC  TTC  CTC  TGT  AAG  CTT  998  999  1000 1001 1002 1003 1004 1005 1006 1007 1008 1009 1010 1011 1012 1013 1014 1015 1016 1017 1018 1019 1020 1021 1022 R    D    T    K    S    T    D    Q    K    M    T    L    L    H    F    L    A    E    L    C    E    N    D    Y    P    CGA  GAC  ACC  AAG  TCC  ACA  GAT  CAG  AAG  ATG  ACG  TTG  TTA  CAC  TTC  TTG  GCT  GAG  TTG  TGT  GAG  AAT  GAC  TAT  CCC  1023 1024 1025 1026 1027 1028 1029 1030 1031 1032 1033 1034 1035 1036 1037 1038 1039 1040 1041 1042 1043 1044 1045 1046 1047 D    V    L    K    F    P    D    E    L    A    H    V    E    K    A    S    R    V    S    A    E    N    L    Q    K    GAT  GTC  CTC  AAG  TTT  CCA  GAC  GAG  CTT  GCC  CAT  GTG  GAG  AAA  GCC  AGC  CGA  GTT  TCT  GCT  GAA  AAC  TTG  CAA  AAG  1048 1049 1050 1051 1052 1053 1054 1055 1056 1057 1058 1059 1060 1061 1062 1063 1064 1065 1066 1067 1068 1069 1070 1071 1072 N    L    D    Q    M    K    K    Q    I    S    D    V    E    R    D    V    Q    N    F    P    A    A    T    D    E    AAC  CTA  GAT  CAG  ATG  AAG  AAA  CAA  ATT  TCT  GAT  GTG  GAA  CGT  GAT  GTT  CAG  AAT  TTC  CCA  GCT  GCC  ACA  GAT  GAA  1073 1074 1075 1076 1077 1078 1079 1080 1081 1082 1083 1084 1085 1086 1087 1088 1089 1090 1091 1092 1093 1094 1095 1096 1097 K    D    K    F    V    E    K    M    T    S    F    V    K    D    A    Q    E    Q    Y    N    K    L    R    M    M    AAA  GAC  AAG  TTT  GTT  GAA  AAA  ATG  ACC  AGC  TTT  GTG  AAG  GAT  GCA  CAG  GAA  CAG  TAT  AAC  AAG  CTG  CGG  ATG  ATG  1098 1099 1100 1101 1102 1103 1104 1105 1106 1107 1108 1109 1110 1111 1112 1113 1114 1115 1116 1117 1118 1119 1120 1121 1122 H    S    N    M    E    T    L    Y    K    E    L    G    E    Y    F    L    F    D    P    K    K    L    S    V    E    CAT  TCT  AAC  ATG  GAG  ACC  CTC  TAT  AAG  GAG  CTG  GGC  GAG  TAC  TTC  CTC  TTT  GAC  CCC  AAG  AAG  TTG  TCT  GTT  GAA  1123 1124 1125 1126 1127 1128 1129 1130 1131 1132 1133 1134 1135 1136 1137 1138 1139 1140 1141 1142 1143 1144 1145 1146 1147 E    F    F    M    D    L    H    N    F    R    N    M    F    L    Q    A    V    K    E    N    Q    K    R    R    E    GAA  TTT  TTC  ATG  GAT  CTT  CAC  AAT  TTT  CGG  AAT  ATG  TTT  TTG  CAA  GCA  GTC  AAG  GAG  AAC  CAG  AAG  CGG  CGG  GAG  1148 1149 1150 1151 1152 1153 1154 1155 1156 1157 1158 1159 1160 1161 1162 1163 1164 1165 1166 1167 1168 1169 1170 1171 1172 T    E    E    K    M    R    R    A    K    L    A    K    E    K    A    E    K    E    R    L    E    K    Q    Q    K    ACA  GAA  GAA  AAG  ATG  AGG  CGA  GCA  AAA  CTA  GCC  AAG  GAG  AAG  GCA  GAG  AAG  GAG  CGG  CTA  GAG  AAG  CAG  CAG  AAG  1173 1174 1175 1176 1177 1178 1179 1180 1181 1182 1183 1184 1185 1186 1187 1188 1189 1190 1191 1192 1193 1194 1195 1196 1197 R    E    Q    L    I    D    M    N    A    E    G    D    E    T    G    V    M    D    S    L    L    E    A    L    Q    AGA  GAG  CAA  CTC  ATA  GAC  ATG  AAT  GCA  GAG  GGC  GAT  GAG  ACA  GGT  GTG  ATG  GAC  AGT  CTT  CTA  GAA  GCC  CTG  CAG  1198 1199 1200 1201 1202 1203 1204 1205 1206 1207 1208 1209 1210 1211 1212 1213 1214 1215 1216 1217 1218 1219 1220 1221 1222 S    G    A    A    F    R    R    K    R    G    P    R    Q    A    N    R    K    A    G    C    A    V    T    S    L    TCA  GGG  GCA  GCA  TTC  CGA  CGG  AAG  AGA  GGG  CCC  CGT  CAA  GCC  AAC  AGG  AAG  GCC  GGG  TGT  GCA  GTC  ACA  TCT  CTG  1223 1224 1225 1226 1227 1228 1229 1230 1231 1232 1233 1234 1235 1236 1237 1238 1239 1240 1241 1242 1243 1244 1245 1246 1247 L    A    S    E    L    T    K    D    D    A    M    A    A    V    P    A    K    V    S    K    N    S    E    T    F    CTA  GCT  TCG  GAG  CTG  ACC  AAG  GAT  GAT  GCC  ATG  GCT  GCT  GTT  CCT  GCC  AAG  GTG  TCC  AAG  AAC  AGT  GAG  ACA  TTC  1248 1249 1250 1251 1252 1253 1254 1255 1256 1257 1258 1259 1260 1261 1262 P    T    I    L    E    E    A    K    E    L    V    G    R    A    S    L    V    P    R    G    S    H    H    H    H    CCC  ACA  ATC  CTT  GAG  GAA  GCC  AAG  GAG  TTG  GTT  GGC  CGT  GCA  AGC  CTG  GTT  CCG  CGT  GGA  TCC  CAC  CAT  CAC  CAT  H   .CAC TGA  GGTDIAPH2-FFC (NP_009293, aa 553–1096)                                                             546  547  548  549  550  551  552  553  554  555  556  557  558  M    G    S    S    H    H    H    H    H    H    G    S    S    G    I    P    G    P    P    A    A    P    P    L    P    ATG  GGC  AGC  AGC  CAT  CAT  CAT  CAT  CAT  CAC  GGA  TCC  TCA  GGA  ATT  CCA  GGT  CCT  CCT  GCA  GCA  CCT  CCA  TTG  CCA  559  560  561  562  563  564  565  566  567  568  569  570  571  572  573  574  575  576  577  578  579  580  581  582  583  G    V    G    P    P    P    P    P    P    A    P    P    L    P    G    G    A    P    L    P    P    P    P    P    P    GGT  GTA  GGG  CCG  CCT  CCA  CCA  CCA  CCC  GCG  CCA  CCT  CTA  CCC  GGA  GGA  GCT  CCT  CTT  CCT  CCT  CCA  CCA  CCT  CCT  584  585  586  587  588  589  590  591  592  593  594  595  596  597  598  599  600  601  602  603  604  605  606  607  608  L    P    G    M    M    G    I    P    P    P    P    P    P    P    L    L    F    G    G    P    P    P    P    P    P    TTA  CCT  GGA  ATG  ATG  GGG  ATA  CCA  CCA  CCA  CCC  CCA  CCA  CCA  CTT  TTA  TTT  GGG  GGA  CCT  CCT  CCA  CCA  CCA  CCC  609  610  611  612  613  614  615  616  617  618  619  620  621  622  623  624  625  626  627  628  629  630  631  632  633  L    G    G    V    P    P    P    P    G    I    S    L    N    L    P    Y    G    M    K    Q    K    K    M    Y    K    CTT  GGA  GGA  GTT  CCT  CCT  CCC  CCA  GGA  ATA  TCA  CTT  AAT  CTA  CCT  TAT  GGA  ATG  AAG  CAG  AAA  AAA  ATG  TAT  AAA  634  635  636  637  638  639  640  641  642  643  644  645  646  647  648  649  650  651  652  653  654  655  656  657  658  P    E    V    S    M    K    R    I    N    W    S    K    I    E    P    T    E    L    S    E    N    C    F    W    L    CCT  GAA  GTG  TCC  ATG  AAG  AGA  ATC  AAT  TGG  TCA  AAG  ATT  GAA  CCC  ACA  GAA  TTA  TCT  GAG  AAC  TGT  TTC  TGG  TTA  659  660  661  662  663  664  665  666  667  668  669  670  671  672  673  674  675  676  677  678  679  680  681  682  683  R    V    K    E    D    K    F    E    N    P    D    L    F    A    K    L    A    L    N    F    A    T    Q    I    K    AGA  GTC  AAA  GAA  GAC  AAG  TTT  GAG  AAT  CCA  GAT  CTC  TTT  GCC  AAA  TTG  GCA  TTG  AAT  TTT  GCT  ACT  CAG  ATA  AAA  684  685  686  687  688  689  690  691  692  693  694  695  696  697  698  699  700  701  702  703  704  705  706  707  708  V    Q    K    N    A    E    A    L    E    E    K    K    T    G    P    T    K    K    K    V    K    E    L    R    I    GTT  CAA  AAG  AAC  GCA  GAA  GCA  TTA  GAA  GAA  AAG  AAG  ACT  GGG  CCT  ACA  AAG  AAG  AAA  GTG  AAA  GAA  CTG  AGA  ATT  709  710  711  712  713  714  715  716  717  718  719  720  721  722  723  724  725  726  727  728  729  730  731  732  733  L    D    P    K    T    A    Q    N    L    S    I    F    L    G    S    Y    R    M    P    Y    E    D    I    R    N    TTG  GAT  CCC  AAA  ACA  GCT  CAG  AAT  CTG  TCC  ATC  TTT  CTG  GGA  TCA  TAT  CGC  ATG  CCA  TAT  GAA  GAC  ATA  AGA  AAC  734  735  736  737  738  739  740  741  742  743  744  745  746  747  748  749  750  751  752  753  754  755  756  757  758  V    I    L    E    V    N    E    D    M    L    S    E    A    L    I    Q    N    L    V    K    H    L    P    E    Q    GTT  ATT  CTG  GAG  GTT  AAT  GAA  GAC  ATG  CTG  AGT  GAG  GCT  TTA  ATT  CAG  AAC  CTT  GTG  AAA  CAT  CTT  CCT  GAG  CAG  759  760  761  762  763  764  765  766  767  768  769  770  771  772  773  774  775  776  777  778  779  780  781  782  783  K    I    L    N    E    L    A    E    L    K    N    E    Y    D    D    L    C    E    P    E    Q    F    G    V    V    AAG  ATA  CTC  AAC  GAA  TTA  GCA  GAG  CTT  AAG  AAT  GAA  TAT  GAT  GAC  CTC  TGT  GAG  CCT  GAA  CAA  TTT  GGA  GTT  GTG  784  785  786  787  788  789  790  791  792  793  794  795  796  797  798  799  800  801  802  803  804  805  806  807  808  M    S    S    V    K    M    L    Q    P    R    L    S    S    I    L    F    K    L    T    F    E    E    H    I    N    ATG  AGC  TCT  GTG  AAA  ATG  TTA  CAG  CCT  CGT  CTC  AGT  AGT  ATC  CTG  TTC  AAG  CTC  ACA  TTT  GAA  GAA  CAC  ATA  AAC  809  810  811  812  813  814  815  816  817  818  819  820  821  822  823  824  825  826  827  828  829  830  831  832  833  N    I    K    P    S    I    I    A    V    T    L    A    C    E    E    L    K    K    S    E    S    F    N    R    L    AAC  ATC  AAA  CCA  AGC  ATC  ATA  GCA  GTA  ACT  CTT  GCC  TGT  GAA  GAA  CTG  AAG  AAA  AGT  GAA  AGC  TTT  AAC  AGA  CTT  834  835  836  837  838  839  840  841  842  843  844  845  846  847  848  849  850  851  852  853  854  855  856  857  858  L    E    L    V    L    L    V    G    N    Y    M    N    S    G    S    R    N    A    Q    S    L    G    F    K    I    TTA  GAG  TTA  GTT  CTT  CTT  GTT  GGA  AAC  TAC  ATG  AAC  TCA  GGC  TCA  AGA  AAT  GCC  CAG  TCT  TTG  GGA  TTT  AAG  ATC  859  860  861  862  863  864  865  866  867  868  869  870  871  872  873  874  875  876  877  878  879  880  881  882  883  N    F    L    C    K    I    R    D    T    K    S    A    D    Q    K    T    T    L    L    H    F    I    A    D    I    AAC  TTC  CTT  TGT  AAG  ATC  AGA  GAT  ACT  AAA  TCA  GCG  GAT  CAA  AAA  ACA  ACC  CTT  TTG  CAT  TTT  ATT  GCC  GAC  ATT  884  885  886  887  888  889  890  891  892  893  894  895  896  897  898  899  900  901  902  903  904  905  906  907  908  C    E    E    K    Y    R    D    I    L    K    F    P    E    E    L    E    H    V    E    S    A    S    K    V    S    TGT  GAG  GAA  AAA  TAT  CGA  GAT  ATC  CTA  AAA  TTT  CCT  GAA  GAA  CTG  GAA  CAC  GTA  GAA  AGT  GCA  AGC  AAA  GTT  TCA  909  910  911  912  913  914  915  916  917  918  919  920  921  922  923  924  925  926  927  928  929  930  931  932  933  A    Q    I    L    K    S    N    L    A    S    M    E    Q    Q    I    V    H    L    E    R    D    I    K    K    F    GCT  CAA  ATT  CTC  AAG  AGC  AAC  CTT  GCA  TCA  ATG  GAA  CAA  CAA  ATT  GTT  CAT  CTG  GAA  CGT  GAC  ATC  AAG  AAA  TTC  934  935  936  937  938  939  940  941  942  943  944  945  946  947  948  949  950  951  952  953  954  955  956  957  958  P    Q    A    E    N    Q    H    D    K    F    V    E    K    M    T    S    F    T    K    T    A    R    E    Q    Y    CCC  CAA  GCA  GAA  AAT  CAA  CAC  GAT  AAG  TTT  GTG  GAA  AAG  ATG  ACC  AGC  TTT  ACA  AAG  ACT  GCC  CGA  GAA  CAG  TAT  959  960  961  962  963  964  965  966  967  968  969  970  971  972  973  974  975  976  977  978  979  980  981  982  983  E    K    L    S    T    M    H    N    N    M    M    K    L    Y    E    N    L    G    E    Y    F    I    F    D    S    GAA  AAA  CTC  TCC  ACC  ATG  CAC  AAC  AAC  ATG  ATG  AAG  CTC  TAT  GAG  AAT  CTT  GGA  GAA  TAC  TTC  ATT  TTT  GAC  TCA  984  985  986  987  988  989  990  991  992  993  994  995  996  997  998  999  1000 1001 1002 1003 1004 1005 1006 1007 1008 K    T    V    S    I    E    E    F    F    G    D    L    N    N    F    R    T    L    F    L    E    A    V    R    E    AAG  ACA  GTG  AGC  ATA  GAA  GAG  TTC  TTT  GGT  GAT  CTC  AAC  AAC  TTC  CGA  ACT  TTG  TTT  TTG  GAA  GCA  GTG  AGA  GAA  1009 1010 1011 1012 1013 1014 1015 1016 1017 1018 1019 1020 1021 1022 1023 1024 1025 1026 1027 1028 1029 1030 1031 1032 1033 N    N    K    R    R    E    M    E    E    K    T    R    R    A    K    L    A    K    E    K    A    E    Q    E    K    AAC  AAT  AAG  AGA  AGA  GAA  ATG  GAA  GAG  AAG  ACC  AGG  AGG  GCA  AAA  CTT  GCA  AAA  GAG  AAA  GCT  GAA  CAA  GAA  AAG  1034 1035 1036 1037 1038 1039 1040 1041 1042 1043 1044 1045 1046 1047 1048 1049 1050 1051 1052 1053 1054 1055 1056 1057 1058 L    E    R    Q    K    K    K    K    Q    L    I    D    I    N    K    E    G    D    E    T    G    V    M    D    N    TTA  GAA  CGC  CAG  AAG  AAA  AAG  AAA  CAA  CTC  ATT  GAT  ATA  AAC  AAA  GAG  GGT  GAT  GAG  ACT  GGT  GTG  ATG  GAT  AAT  1059 1060 1061 1062 1063 1064 1065 1066 1067 1068 1069 1070 1071 1072 1073 1074 1075 1076 1077 1078 1079 1080 1081 1082 1083 L    L    E    A    L    Q    S    G    A    A    F    R    D    R    R    K    R    I    P    R    N    P    V    V    N    CTT  CTA  GAA  GCC  CTA  CAA  TCA  GGT  GCA  GCA  TTC  AGA  GAC  CGT  CGA  AAG  CGG  ATT  CCA  AGG  AAT  CCA  GTG  GTA  AAT  1084 1085 1086 1087 1088 1089 1090 1091 1092 1093 1094 1095 1096 H    P    C    A    T    R    A    N    P    R    S    A    T    .    CAT  CCC  TGT  GCA  ACA  AGG  GCT  AAT  CCA  AGA  TCA  GCT  ACA  TAA  GGTACCCCGGCT                                     FMNL3-FFC (NP_001354764.1, aa 481-1028)                                                            481  482  483  484  485  486  487  488  489  490  491  492  493  M    G    S    S    H    H    H    H    H    H    G    S    V    D    S    E    A    L    A    R    V    G    P    A    E    ATG  GGC  AGC  AGC  CAT  CAT  CAT  CAT  CAT  CAC  GGA  TCC  GTA  GAT  AGT  GAG  GCA  CTG  GCC  CGC  GTG  GGC  CCT  GCA  GAA  494  495  496  497  498  499  500  501  502  503  504  505  506  507  508  509  510  511  512  513  514  515  516  517  518  L    S    E    G    M    P    P    S    D    L    D    L    L    A    P    A    P    P    P    E    E    V    L    P    L    TTG  TCT  GAG  GGA  ATG  CCC  CCT  TCT  GAC  CTG  GAC  CTG  TTA  GCC  CCA  GCA  CCG  CCC  CCG  GAA  GAG  GTG  TTA  CCC  CTT  519  520  521  522  523  524  525  526  527  528  529  530  531  532  533  534  535  536  537  538  539  540  541  542  543  P    P    P    P    A    P    P    L    P    P    P    P    P    P    L    P    D    K    C    P    P    A    P    P    L    CCA  CCT  CCC  CCG  GCT  CCA  CCC  TTG  CCT  CCG  CCG  CCC  CCA  CCT  CTT  CCG  GAT  AAA  TGC  CCG  CCT  GCG  CCC  CCC  CTT  544  545  546  547  548  549  550  551  552  553  554  555  556  557  558  559  560  561  562  563  564  565  566  567  568  P    G    A    A    P    S    V    V    L    T    V    G    L    S    A    I    R    I    K    K    P    I    K    T    K    CCG  GGT  GCC  GCA  CCA  AGT  GTT  GTG  TTG  ACT  GTC  GGA  TTG  TCC  GCC  ATC  CGT  ATC  AAA  AAA  CCC  ATC  AAA  ACC  AAG  569  570  571  572  573  574  575  576  577  578  579  580  581  582  583  584  585  586  587  588  589  590  591  592  593  F    R    L    P    V    F    N    W    T    A    L    K    P    N    Q    I    S    G    T    V    F    S    E    L    D    TTC  CGT  CTG  CCC  GTT  TTC  AAC  TGG  ACC  GCA  TTA  AAG  CCG  AAT  CAA  ATC  TCT  GGG  ACA  GTT  TTC  AGT  GAA  CTT  GAT  594  595  596  597  598  599  600  601  602  603  604  605  606  607  608  609  610  611  612  613  614  615  616  617  618  D    E    K    I    L    E    D    L    D    L    D    K    F    E    E    L    F    K    T    K    A    Q    G    P    A    GAC  GAA  AAG  ATT  CTG  GAG  GAC  CTG  GAC  CTG  GAC  AAA  TTT  GAA  GAA  CTG  TTT  AAG  ACG  AAA  GCG  CAA  GGC  CCA  GCA  619  620  621  622  623  624  625  626  627  628  629  630  631  632  633  634  635  636  637  638  639  640  641  642  643  L    D    L    I    C    S    K    N    K    T    A    Q    K    A    A    S    K    V    T    L    L    E    A    N    R    CTG  GAC  TTG  ATC  TGC  AGC  AAG  AAC  AAA  ACT  GCC  CAA  AAG  GCG  GCG  TCA  AAA  GTC  ACA  CTT  TTG  GAA  GCA  AAT  CGC  644  645  646  647  648  649  650  651  652  653  654  655  656  657  658  659  660  661  662  663  664  665  666  667  668  A    K    N    L    A    I    T    L    R    K    A    G    R    S    A    E    E    I    C    R    A    I    H    T    F    GCG  AAG  AAC  CTT  GCC  ATT  ACA  CTT  CGT  AAG  GCT  GGT  CGC  TCG  GCT  GAA  GAG  ATC  TGT  CGT  GCC  ATC  CAT  ACC  TTC  669  670  671  672  673  674  675  676  677  678  679  680  681  682  683  684  685  686  687  688  689  690  691  692  693  D    L    Q    T    L    P    V    D    F    V    E    C    L    M    R    F    L    P    T    E    A    E    V    K    L    GAC  CTG  CAG  ACA  CTG  CCA  GTA  GAC  TTC  GTG  GAA  TGC  TTG  ATG  CGT  TTT  CTT  CCG  ACG  GAA  GCG  GAA  GTT  AAG  TTG  694  695  696  697  698  699  700  701  702  703  704  705  706  707  708  709  710  711  712  713  714  715  716  717  718  L    R    Q    Y    E    R    E    R    Q    P    L    E    E    L    A    A    E    D    R    F    M    L    L    F    S    TTG  CGC  CAG  TAC  GAG  CGT  GAG  CGT  CAG  CCC  CTT  GAG  GAA  TTA  GCC  GCT  GAA  GAC  CGC  TTC  ATG  TTA  CTG  TTC  TCG  719  720  721  722  723  724  725  726  727  728  729  730  731  732  733  734  735  736  737  738  739  740  741  742  743  K    V    E    R    L    T    Q    R    M    A    G    M    A    F    L    G    N    F    Q    D    N    L    Q    M    L    AAG  GTC  GAA  CGC  TTG  ACG  CAA  CGC  ATG  GCA  GGC  ATG  GCT  TTC  TTG  GGT  AAT  TTT  CAA  GAC  AAT  TTG  CAA  ATG  TTA  744  745  746  747  748  749  750  751  752  753  754  755  756  757  758  759  760  761  762  763  764  765  766  767  768  T    P    Q    L    N    A    I    I    A    A    S    A    S    V    K    S    S    Q    K    L    K    Q    M    L    E    ACT  CCT  CAG  CTT  AAT  GCG  ATT  ATT  GCA  GCC  TCC  GCA  TCT  GTC  AAG  AGC  TCG  CAA  AAG  CTG  AAA  CAG  ATG  TTA  GAA  769  770  771  772  773  774  775  776  777  778  779  780  781  782  783  784  785  786  787  788  789  790  791  792  793  I    I    L    A    L    G    N    Y    M    N    S    S    K    R    G    A    V    Y    G    F    K    L    Q    S    L    ATT  ATT  CTG  GCC  TTA  GGG  AAT  TAT  ATG  AAC  TCC  AGT  AAA  CGT  GGT  GCT  GTC  TAT  GGT  TTC  AAG  CTT  CAA  TCA  TTA  794  795  796  797  798  799  800  801  802  803  804  805  806  807  808  809  810  811  812  813  814  815  816  817  818  D    L    L    L    D    T    K    S    T    D    R    K    M    T    L    L    H    F    I    A    L    T    V    K    E    GAC  CTT  CTT  CTT  GAT  ACC  AAA  TCC  ACG  GAC  CGC  AAA  ATG  ACG  TTG  CTG  CAT  TTC  ATT  GCA  TTG  ACA  GTT  AAA  GAA  819  820  821  822  823  824  825  826  827  828  829  830  831  832  833  834  835  836  837  838  839  840  841  842  843  K    Y    P    D    L    A    N    F    W    H    E    L    H    F    V    E    K    A    A    A    V    S    L    E    N    AAG  TAT  CCC  GAC  TTG  GCG  AAC  TTC  TGG  CAC  GAG  CTG  CAT  TTC  GTA  GAG  AAG  GCT  GCA  GCC  GTC  TCC  CTT  GAG  AAT  844  845  846  847  848  849  850  851  852  853  854  855  856  857  858  859  860  861  862  863  864  865  866  867  868  V    L    L    D    V    K    E    L    G    R    G    M    E    L    I    R    R    E    C    S    I    H    D    N    S    GTT  CTT  TTA  GAC  GTC  AAG  GAG  TTA  GGA  CGT  GGG  ATG  GAA  CTT  ATC  CGT  CGC  GAG  TGT  AGT  ATT  CAT  GAT  AAC  AGC  869  870  871  872  873  874  875  876  877  878  879  880  881  882  883  884  885  886  887  888  889  890  891  892  893  V    L    R    N    F    L    S    T    N    E    G    K    L    D    K    L    Q    R    D    A    K    T    A    E    E    GTG  CTT  CGT  AAC  TTC  TTG  TCA  ACC  AAC  GAA  GGC  AAG  CTG  GAT  AAG  TTA  CAA  CGT  GAT  GCG  AAA  ACG  GCG  GAA  GAA  894  895  896  897  898  899  900  901  902  903  904  905  906  907  908  909  910  911  912  913  914  915  916  917  918  A    Y    N    A    V    V    R    Y    F    G    E    S    P    K    T    T    P    P    S    V    F    F    P    V    F    GCG  TAT  AAC  GCG  GTT  GTG  CGT  TAC  TTC  GGA  GAA  TCC  CCT  AAG  ACA  ACG  CCT  CCC  TCA  GTC  TTC  TTT  CCT  GTC  TTT  919  920  921  922  923  924  925  926  927  928  929  930  931  932  933  934  935  936  937  938  939  940  941  942  943  V    R    F    I    R    S    Y    K    E    A    E    Q    E    N    E    A    R    K    K    Q    E    E    V    M    R    GTT  CGT  TTT  ATT  CGC  AGT  TAC  AAA  GAG  GCA  GAA  CAG  GAG  AAT  GAA  GCC  CGC  AAG  AAG  CAA  GAA  GAA  GTA  ATG  CGT  944  945  946  947  948  949  950  951  952  953  954  955  956  957  958  959  960  961  962  963  964  965  966  967  968  E    K    Q    L    A    Q    E    A    K    K    L    D    A    K    T    P    S    Q    R    N    K    W    Q    Q    Q    GAG  AAA  CAA  TTA  GCA  CAA  GAG  GCA  AAG  AAA  TTG  GAT  GCC  AAG  ACC  CCT  TCA  CAA  CGC  AAC  AAA  TGG  CAG  CAG  CAG  969  970  971  972  973  974  975  976  977  978  979  980  981  982  983  984  985  986  987  988  989  990  991  992  993  E    L    I    A    E    L    R    R    R    Q    A    K    E    H    R    P    V    Y    E    G    K    D    G    T    I    GAG  TTG  ATC  GCT  GAG  CTG  CGT  CGC  CGT  CAG  GCG  AAG  GAA  CAT  CGT  CCA  GTT  TAC  GAG  GGA  AAA  GAC  GGC  ACT  ATT  994  995  996  997  998  999  1000 1001 1002 1003 1004 1005 1006 1007 1008 1009 1010 1011 1012 1013 1014 1015 1016 1017 1018 E    D    I    I    T    V    L    K    S    V    P    F    T    A    R    T    A    K    R    G    S    R    F    F    C    GAG  GAC  ATC  ATC  ACG  GTT  CTG  AAA  TCA  GTA  CCT  TTT  ACA  GCC  CGC  ACC  GCG  AAG  CGC  GGG  TCA  CGC  TTC  TTT  TGT  1019 1020 1021 1022 1023 1024 1025 1026 1027 1028 D    A    A    H    H    D    E    S    N    C    .    GAC  GCC  GCA  CAC  CAT  GAT  GAA  AGC  AAC  TGT  TGA  GGTACCCCGGCTGCTAAC                                        FMN2-FFC (NP_064450.3, aa 1192-1722)                                                            1192 1193 1194 1195 1196 1197 1198 1199 1200 1201 1202 1203 1204 M    G    S    S    H    H    H    H    H    H    G    S    G    V    G    I    P    P    P    P    P    L    P    G    A    ATG  GGC  AGC  AGC  CAT  CAT  CAT  CAT  CAT  CAC  GGA  TCC  GGG  GTC  GGA  ATT  CCT  CCT  CCC  CCA  CCA  TTA  CCT  GGA  GCC  1205 1206 1207 1208 1209 1210 1211 1212 1213 1214 1215 1216 1217 1218 1219 1220 1221 1222 1223 1224 1225 1226 1227 1228 1229 G    I    P    P    P    P    P    L    P    G    M    G    I    P    P    A    P    A    P    P    L    P    P    P    G    GGC  ATC  CCT  CCA  CCT  CCT  CCT  TTG  CCA  GGT  ATG  GGA  ATT  CCT  CCC  GCA  CCC  GCT  CCA  CCT  CTT  CCT  CCT  CCG  GGA  1230 1231 1232 1233 1234 1235 1236 1237 1238 1239 1240 1241 1242 1243 1244 1245 1246 1247 1248 1249 1250 1251 1252 1253 1254 T    G    I    P    P    P    P    L    L    P    V    S    G    P    P    L    L    P    Q    V    G    S    S    T    L    ACT  GGA  ATT  CCG  CCA  CCA  CCA  CTT  TTA  CCT  GTC  AGT  GGG  CCC  CCA  TTA  TTA  CCT  CAG  GTA  GGA  AGC  TCC  ACA  CTT  1255 1256 1257 1258 1259 1260 1261 1262 1263 1264 1265 1266 1267 1268 1269 1270 1271 1272 1273 1274 1275 1276 1277 1278 1279 P    T    P    Q    V    C    G    F    L    P    P    P    L    P    S    G    L    F    G    L    G    M    N    Q    D    CCA  ACG  CCA  CAG  GTT  TGT  GGT  TTT  CTG  CCC  CCA  CCG  TTG  CCC  TCT  GGC  TTG  TTT  GGG  TTA  GGC  ATG  AAC  CAA  GAC  1280 1281 1282 1283 1284 1285 1286 1287 1288 1289 1290 1291 1292 1293 1294 1295 1296 1297 1298 1299 1300 1301 1302 1303 1304 K    G    S    R    K    Q    P    I    E    P    C    R    P    M    K    P    L    Y    W    T    R    I    Q    L    H    AAG  GGC  TCG  CGC  AAG  CAA  CCA  ATT  GAA  CCG  TGC  CGT  CCA  ATG  AAG  CCG  CTG  TAC  TGG  ACC  CGC  ATC  CAG  TTG  CAC  1305 1306 1307 1308 1309 1310 1311 1312 1313 1314 1315 1316 1317 1318 1319 1320 1321 1322 1323 1324 1325 1326 1327 1328 1329 S    K    R    D    S    S    T    S    L    I    W    E    K    I    E    E    P    S    I    D    C    H    E    F    E    TCC  AAG  CGC  GAC  TCT  TCA  ACG  TCT  TTA  ATC  TGG  GAA  AAA  ATT  GAA  GAG  CCA  AGT  ATC  GAC  TGC  CAT  GAA  TTT  GAA  1330 1331 1332 1333 1334 1335 1336 1337 1338 1339 1340 1341 1342 1343 1344 1345 1346 1347 1348 1349 1350 1351 1352 1353 1354 E    L    F    S    K    T    A    V    K    E    R    K    K    P    I    S    D    T    I    S    K    T    K    A    K    GAA  TTA  TTT  TCC  AAG  ACC  GCA  GTC  AAG  GAG  CGT  AAG  AAG  CCT  ATC  TCC  GAT  ACG  ATT  TCG  AAG  ACA  AAA  GCA  AAA  1355 1356 1357 1358 1359 1360 1361 1362 1363 1364 1365 1366 1367 1368 1369 1370 1371 1372 1373 1374 1375 1376 1377 1378 1379 Q    V    V    K    L    L    S    N    K    R    S    Q    A    V    G    I    L    M    S    S    L    H    L    D    M    CAA  GTG  GTC  AAA  TTA  CTT  TCT  AAT  AAA  CGT  TCC  CAG  GCT  GTC  GGG  ATC  TTG  ATG  TCG  TCC  TTA  CAC  CTG  GAT  ATG  1380 1381 1382 1383 1384 1385 1386 1387 1388 1389 1390 1391 1392 1393 1394 1395 1396 1397 1398 1399 1400 1401 1402 1403 1404 K    D    I    Q    H    A    V    V    N    L    D    N    S    V    V    D    L    E    T    L    Q    A    L    Y    E    AAA  GAC  ATT  CAA  CAT  GCG  GTG  GTT  AAT  CTG  GAC  AAT  AGT  GTG  GTG  GAC  TTG  GAA  ACT  CTT  CAA  GCG  TTG  TAT  GAA  1405 1406 1407 1408 1409 1410 1411 1412 1413 1414 1415 1416 1417 1418 1419 1420 1421 1422 1423 1424 1425 1426 1427 1428 1429 N    R    A    Q    S    D    E    L    E    K    I    E    K    H    G    R    S    S    K    D    K    E    N    A    K    AAC  CGC  GCG  CAA  TCT  GAC  GAG  TTG  GAA  AAA  ATT  GAG  AAA  CAT  GGA  CGT  TCT  AGC  AAG  GAC  AAG  GAA  AAC  GCG  AAA  1430 1431 1432 1433 1434 1435 1436 1437 1438 1439 1440 1441 1442 1443 1444 1445 1446 1447 1448 1449 1450 1451 1452 1453 1454 S    L    D    K    P    E    Q    F    L    Y    E    L    S    L    I    P    N    F    S    E    R    V    F    C    I    TCT  TTA  GAC  AAA  CCG  GAG  CAA  TTC  TTA  TAC  GAG  CTT  TCT  CTG  ATT  CCG  AAC  TTT  TCC  GAA  CGC  GTT  TTT  TGT  ATC  1455 1456 1457 1458 1459 1460 1461 1462 1463 1464 1465 1466 1467 1468 1469 1470 1471 1472 1473 1474 1475 1476 1477 1478 1479 L    F    Q    S    T    F    S    E    S    I    C    S    I    R    R    K    L    E    L    L    Q    K    L    C    E    TTG  TTT  CAG  TCA  ACT  TTT  AGC  GAA  AGT  ATC  TGT  TCG  ATC  CGC  CGC  AAA  TTG  GAG  TTG  CTT  CAG  AAG  CTG  TGC  GAG  1480 1481 1482 1483 1484 1485 1486 1487 1488 1489 1490 1491 1492 1493 1494 1495 1496 1497 1498 1499 1500 1501 1502 1503 1504 T    L    K    N    G    P    G    V    M    Q    V    L    G    L    V    L    A    F    G    N    Y    M    N    G    G    ACT  CTT  AAA  AAC  GGG  CCG  GGC  GTC  ATG  CAG  GTC  TTG  GGA  CTT  GTC  TTG  GCT  TTT  GGC  AAT  TAC  ATG  AAC  GGT  GGG  1505 1506 1507 1508 1509 1510 1511 1512 1513 1514 1515 1516 1517 1518 1519 1520 1521 1522 1523 1524 1525 1526 1527 1528 1529 N    K    T    R    G    Q    A    D    G    F    G    L    D    I    L    P    K    L    K    D    V    K    S    S    D    AAT  AAG  ACT  CGC  GGG  CAA  GCG  GAC  GGA  TTT  GGT  TTG  GAT  ATC  CTT  CCT  AAG  CTG  AAG  GAC  GTT  AAG  TCC  TCC  GAT  1530 1531 1532 1533 1534 1535 1536 1537 1538 1539 1540 1541 1542 1543 1544 1545 1546 1547 1548 1549 1550 1551 1552 1553 1554 N    S    R    S    L    L    S    Y    I    V    S    Y    Y    L    R    N    F    D    E    D    A    G    K    E    Q    AAC  TCT  CGT  AGT  CTG  CTT  TCC  TAC  ATT  GTT  AGT  TAC  TAC  TTA  CGT  AAT  TTC  GAT  GAG  GAT  GCT  GGA  AAG  GAA  CAG  1555 1556 1557 1558 1559 1560 1561 1562 1563 1564 1565 1566 1567 1568 1569 1570 1571 1572 1573 1574 1575 1576 1577 1578 1579 C    L    F    P    L    P    E    P    Q    D    L    F    Q    A    S    Q    M    K    F    E    D    F    Q    K    D    TGC  TTG  TTT  CCC  TTG  CCA  GAG  CCA  CAA  GAC  CTT  TTT  CAG  GCA  AGC  CAG  ATG  AAG  TTT  GAA  GAC  TTT  CAA  AAA  GAT  1580 1581 1582 1583 1584 1585 1586 1587 1588 1589 1590 1591 1592 1593 1594 1595 1596 1597 1598 1599 1600 1601 1602 1603 1604 L    R    K    L    K    K    D    L    K    A    C    E    V    E    A    G    K    V    Y    Q    V    S    S    K    E    TTG  CGT  AAA  TTA  AAA  AAG  GAT  TTG  AAA  GCT  TGC  GAG  GTG  GAG  GCA  GGG  AAA  GTA  TAC  CAA  GTT  TCA  AGT  AAG  GAG  1605 1606 1607 1608 1609 1610 1611 1612 1613 1614 1615 1616 1617 1618 1619 1620 1621 1622 1623 1624 1625 1626 1627 1628 1629 H    M    Q    P    F    K    E    N    M    E    Q    F    I    I    Q    A    K    I    D    Q    E    A    E    E    N    CAT  ATG  CAG  CCG  TTC  AAA  GAG  AAC  ATG  GAG  CAA  TTT  ATT  ATT  CAA  GCT  AAG  ATT  GAC  CAA  GAG  GCA  GAA  GAG  AAC  1630 1631 1632 1633 1634 1635 1636 1637 1638 1639 1640 1641 1642 1643 1644 1645 1646 1647 1648 1649 1650 1651 1652 1653 1654 S    L    T    E    T    H    K    C    F    L    E    T    T    A    Y    F    F    M    K    P    K    L    G    E    K    AGT  TTA  ACA  GAA  ACT  CAT  AAA  TGT  TTC  CTG  GAG  ACT  ACG  GCC  TAC  TTT  TTT  ATG  AAG  CCC  AAA  CTT  GGT  GAG  AAG  1655 1656 1657 1658 1659 1660 1661 1662 1663 1664 1665 1666 1667 1668 1669 1670 1671 1672 1673 1674 1675 1676 1677 1678 1679 E    V    S    P    N    A    F    F    S    I    W    H    E    F    S    S    D    F    K    D    F    W    K    K    E    GAG  GTT  TCT  CCA  AAC  GCA  TTT  TTC  AGT  ATT  TGG  CAT  GAG  TTC  TCT  TCA  GAC  TTC  AAA  GAT  TTT  TGG  AAG  AAG  GAA  1680 1681 1682 1683 1684 1685 1686 1687 1688 1689 1690 1691 1692 1693 1694 1695 1696 1697 1698 1699 1700 1701 1702 1703 1704 N    K    L    L    L    Q    E    R    V    K    E    A    E    E    V    C    R    Q    K    K    G    K    S    L    Y    AAT  AAA  CTT  CTT  CTG  CAA  GAG  CGT  GTT  AAA  GAG  GCA  GAG  GAA  GTC  TGC  CGC  CAA  AAA  AAA  GGT  AAA  TCA  CTT  TAC  1705 1706 1707 1708 1709 1710 1711 1712 1713 1714 1715 1716 1717 1718 1719 1720 1721 1722 K    I    K    P    R    H    D    S    G    I    K    A    K    I    S    M    K    T    .    AAA  ATT  AAA  CCC  CGT  CAT  GAC  TCC  GGT  ATC  AAA  GCA  AAG  ATT  TCA  ATG  AAA  ACT  TGA  GGTACCCCGGCT            Delphilin-FFC (NP_001138590, aa 744-1211)                                                            794  795  796  797  798  799  800  801  802  803  804  805  806  M    G    S    S    H    H    H    H    H    H    G    S    S    I    S    D    H    I    P    P    P    P    L    S    P    ATG  GGC  AGC  AGC  CAT  CAT  CAT  CAT  CAT  CAC  GGA  TCC  TCC  ATC  TCT  GAT  CAC  ATT  CCT  CCA  CCT  CCC  CTT  TCT  CCA  807  808  809  810  811  812  813  814  815  816  817  818  819  820  821  822  823  824  825  826  827  828  829  830  831  P    P    P    P    P    L    P    F    H    D    A    K    P    S    S    R    S    S    D    G    S    R    G    P    A    CCT  CCT  CCT  CCG  CCG  CTT  CCT  TTT  CAT  GAC  GCA  AAA  CCT  TCG  AGT  CGT  TCC  TCA  GAC  GGG  TCA  CGC  GGC  CCA  GCG  832  833  834  835  836  837  838  839  840  841  842  843  844  845  846  847  848  849  850  851  852  853  854  855  856  Q    A    L    A    K    P    L    T    Q    L    S    H    P    V    P    P    P    P    P    P    P    L    P    P    P    CAA  GCT  TTG  GCA  AAA  CCT  TTG  ACG  CAG  TTG  TCC  CAC  CCA  GTC  CCA  CCT  CCG  CCC  CCG  CCG  CCG  CTG  CCG  CCC  CCG  857  858  859  860  861  862  863  864  865  866  867  868  869  870  871  872  873  874  875  876  877  878  879  880  881  V    P    C    A    P    P    M    L    S    R    G    L    G    H    R    R    S    E    T    S    H    M    S    V    K    GTC  CCT  TGC  GCA  CCG  CCT  ATG  TTG  AGC  CGT  GGC  CTT  GGT  CAC  CGC  CGC  TCC  GAA  ACT  TCT  CAC  ATG  TCC  GTA  AAG  882  883  884  885  886  887  888  889  890  891  892  893  894  895  896  897  898  899  900  901  902  903  904  905  906  R    L    R    W    E    Q    V    E    N    S    E    G    T    I    W    G    Q    L    G    E    D    S    D    Y    D    CGC  CTG  CGT  TGG  GAG  CAG  GTC  GAG  AAT  TCC  GAA  GGC  ACT  ATT  TGG  GGA  CAG  TTA  GGC  GAG  GAC  TCC  GAT  TAT  GAC  907  908  909  910  911  912  913  914  915  916  917  918  919  920  921  922  923  924  925  926  927  928  929  930  931  K    L    S    D    M    V    K    Y    L    D    L    E    L    H    F    G    T    Q    K    P    A    K    P    V    P    AAA  CTG  TCA  GAT  ATG  GTG  AAA  TAT  CTG  GAC  CTG  GAA  CTG  CAT  TTT  GGT  ACC  CAA  AAG  CCG  GCT  AAA  CCC  GTC  CCG  932  933  934  935  936  937  938  939  940  941  942  943  944  945  946  947  948  949  950  951  952  953  954  955  956  G    P    E    P    F    R    K    K    E    V    V    E    I    L    S    H    K    K    A    Y    N    T    S    I    L    GGT  CCC  GAG  CCT  TTT  CGC  AAG  AAG  GAG  GTA  GTG  GAA  ATC  CTG  AGT  CAC  AAG  AAG  GCG  TAC  AAC  ACC  TCG  ATT  TTA  957  958  959  960  961  962  963  964  965  966  967  968  969  970  971  972  973  974  975  976  977  978  979  980  981  L    A    H    L    K    L    S    P    A    E    L    R    Q    V    L    M    S    M    E    P    R    R    L    E    P    TTG  GCG  CAC  CTG  AAA  CTG  TCG  CCC  GCG  GAA  TTA  CGT  CAG  GTA  TTA  ATG  TCA  ATG  GAA  CCC  CGT  CGC  TTG  GAG  CCT  982  983  984  985  986  987  988  989  990  991  992  993  994  995  996  997  998  999  1000 1001 1002 1003 1004 1005 1006 A    H    L    A    Q    L    L    L    F    A    P    D    A    D    E    E    Q    R    Y    Q    A    F    R    E    A    GCG  CAC  CTT  GCA  CAA  TTA  TTG  TTG  TTT  GCA  CCT  GAT  GCT  GAC  GAG  GAA  CAA  CGC  TAT  CAG  GCT  TTC  CGT  GAG  GCA  1007 1008 1009 1010 1011 1012 1013 1014 1015 1016 1017 1018 1019 1020 1021 1022 1023 1024 1025 1026 1027 1028 1029 1030 1031 P    G    R    L    S    E    P    D    Q    F    V    L    Q    M    L    S    V    P    E    Y    K    T    R    L    R    CCC  GGA  CGT  CTG  TCG  GAA  CCT  GAC  CAA  TTC  GTT  TTA  CAG  ATG  CTT  TCT  GTA  CCG  GAA  TAT  AAA  ACT  CGT  TTG  CGT  1032 1033 1034 1035 1036 1037 1038 1039 1040 1041 1042 1043 1044 1045 1046 1047 1048 1049 1050 1051 1052 1053 1054 1055 1056 S    L    H    F    Q    A    T    L    Q    E    K    T    E    E    I    R    G    S    L    E    C    L    R    Q    A    TCA  CTG  CAC  TTC  CAG  GCA  ACA  CTG  CAG  GAG  AAG  ACT  GAG  GAG  ATT  CGT  GGA  TCG  TTG  GAA  TGT  TTG  CGT  CAG  GCA  1057 1058 1059 1060 1061 1062 1063 1064 1065 1066 1067 1068 1069 1070 1071 1072 1073 1074 1075 1076 1077 1078 1079 1080 1081 S    L    E    L    K    N    S    R    K    L    A    K    I    L    E    F    V    L    A    M    G    N    Y    L    N    AGC  TTA  GAA  TTA  AAG  AAC  AGT  CGC  AAA  CTT  GCG  AAG  ATC  CTG  GAA  TTC  GTG  CTT  GCT  ATG  GGC  AAC  TAT  CTT  AAC  1082 1083 1084 1085 1086 1087 1088 1089 1090 1091 1092 1093 1094 1095 1096 1097 1098 1099 1100 1101 1102 1103 1104 1105 1106 D    G    Q    P    K    T    N    K    T    T    G    F    K    I    N    F    L    T    E    L    N    S    T    K    T    GAT  GGT  CAG  CCT  AAA  ACA  AAC  AAA  ACC  ACG  GGC  TTT  AAG  ATC  AAC  TTT  TTA  ACG  GAG  TTA  AAC  TCT  ACC  AAA  ACT  1107 1108 1109 1110 1111 1112 1113 1114 1115 1116 1117 1118 1119 1120 1121 1122 1123 1124 1125 1126 1127 1128 1129 1130 1131 V    D    G    K    S    T    F    L    H    I    L    A    K    S    L    S    Q    H    F    P    E    L    L    G    F    GTT  GAC  GGC  AAA  AGT  ACT  TTC  TTG  CAT  ATT  CTG  GCC  AAG  AGC  CTG  AGC  CAG  CAC  TTC  CCG  GAA  TTG  TTA  GGT  TTC  1132 1133 1134 1135 1136 1137 1138 1139 1140 1141 1142 1143 1144 1145 1146 1147 1148 1149 1150 1151 1152 1153 1154 1155 1156 A    Q    D    L    P    T    V    P    L    A    A    K    V    N    Q    R    A    L    T    S    D    L    A    D    L    GCA  CAG  GAC  TTA  CCT  ACT  GTG  CCG  CTT  GCT  GCG  AAG  GTG  AAC  CAA  CGC  GCT  TTA  ACG  TCG  GAC  TTA  GCG  GAC  TTA  1157 1158 1159 1160 1161 1162 1163 1164 1165 1166 1167 1168 1169 1170 1171 1172 1173 1174 1175 1176 1177 1178 1179 1180 1181 H    G    T    I    S    E    I    Q    D    A    C    Q    S    I    S    P    S    S    E    D    K    F    A    M    V    CAC  GGG  ACT  ATT  TCG  GAG  ATT  CAA  GAT  GCG  TGT  CAG  AGC  ATC  TCA  CCC  AGC  TCG  GAA  GAC  AAA  TTT  GCC  ATG  GTA  1182 1183 1184 1185 1186 1187 1188 1189 1190 1191 1192 1193 1194 1195 1196 1197 1198 1199 1200 1201 1202 1203 1204 1205 1206 M    S    S    F    L    E    T    A    Q    P    A    L    R    A    L    D    G    L    Q    R    E    A    M    E    E    ATG  AGC  AGC  TTT  CTG  GAA  ACA  GCC  CAA  CCC  GCT  TTG  CGC  GCC  CTT  GAT  GGC  CTT  CAG  CGC  GAA  GCT  ATG  GAG  GAA  1207 1208 1209 1210 1211 1212 1213 1214 1215 1216 1217 1218 1219 1220 1221 1222 1223 1224 1225 1226 1227 1228 1229 1230 1231 L    G    K    A    L    A    F    F    G    E    D    S    K    A    T    T    S    E    A    F    F    G    I    F    A    TTG  GGC  AAG  GCA  TTA  GCG  TTT  TTC  GGC  GAG  GAC  AGC  AAG  GCG  ACA  ACT  TCC  GAA  GCG  TTC  TTT  GGC  ATC  TTT  GCT  1232 1233 1234 1235 1236 1237 1238 1239 1240 1241 1242 1243 1244 1245 1246 1247 1248 1249 1250 1251 1252 1253 1254 1255 1256 E    F    M    S    K    F    E    R    A    L    S    D    L    Q    A    G    E    G    L    R    S    S    G    M    V    GAA  TTT  ATG  TCA  AAG  TTC  GAA  CGC  GCA  TTG  TCC  GAT  TTG  CAA  GCA  GGA  GAG  GGG  TTG  CGC  TCC  TCA  GGG  ATG  GTA  1257 1258 1259 1260 1261 S    P    L    A    W    .    TCA  CCA  TTG  GCC  TGG  TGA  GGTACCCCGGCTGCTAAC                                                                   INF2-FFC (NP_071934.3, aa 469–1249)                                                            469  470  471  472  473  474  475  476  477  478  479  480  481  M    G    S    S    H    H    H    H    H    H    G    S    M    A    P    P    A    P    P    L    P    P    P    L    P    ATG  GGC  AGC  AGC  CAT  CAT  CAT  CAT  CAT  CAC  GGA  TCC  ATG  GCC  CCC  CCA  GCA  CCT  CCT  CTA  CCA  CCA  CCC  CTG  CCA  482  483  484  485  486  487  488  489  490  491  492  493  494  495  496  497  498  499  500  501  502  503  504  505  506  G    S    C    E    F    L    P    P    P    P    P    P    L    P    G    L    G    C    P    P    P    P    P    P    L    GGC  TCC  TGT  GAG  TTC  CTG  CCC  CCA  CCA  CCT  CCA  CCA  CTC  CCG  GGC  TTG  GGA  TGC  CCG  CCC  CCA  CCC  CCA  CCC  CTG  507  508  509  510  511  512  513  514  515  516  517  518  519  520  521  522  523  524  525  526  527  528  529  530  531  L    P    G    M    G    W    G    P    P    P    P    P    P    P    L    L    P    C    T    C    S    P    P    V    A    CTG  CCT  GGT  ATG  GGC  TGG  GGC  CCT  CCT  CCA  CCC  CCA  CCT  CCA  CTA  CTG  CCC  TGC  ACC  TGC  AGC  CCC  CCC  GTG  GCG  532  533  534  535  536  537  538  539  540  541  542  543  544  545  546  547  548  549  550  551  552  553  554  555  556  G    G    M    E    E    V    I    V    A    Q    V    D    H    G    L    G    S    A    W    V    P    S    H    R    R    GGA  GGC  ATG  GAG  GAG  GTC  ATC  GTG  GCC  CAG  GTG  GAC  CAT  GGC  TTG  GGC  TCA  GCA  TGG  GTC  CCC  AGC  CAT  CGG  CGG  557  558  559  560  561  562  563  564  565  566  567  568  569  570  571  572  573  574  575  576  577  578  579  580  581  V    N    P    P    T    L    R    M    K    K    L    N    W    Q    K    L    P    S    N    V    A    R    E    H    N    GTG  AAC  CCA  CCC  ACA  CTG  CGC  ATG  AAG  AAG  CTG  AAC  TGG  CAG  AAG  CTG  CCA  TCC  AAC  GTG  GCA  CGT  GAG  CAC  AAC  582  583  584  585  586  587  588  589  590  591  592  593  594  595  596  597  598  599  600  601  602  603  604  605  606  S    M    W    A    S    L    S    S    P    D    A    E    A    V    E    P    D    F    S    S    I    E    R    L    F    TCT  ATG  TGG  GCG  TCC  CTG  AGC  AGC  CCC  GAC  GCC  GAG  GCT  GTG  GAG  CCC  GAC  TTC  TCC  AGC  ATC  GAG  CGA  CTA  TTC  607  608  609  610  611  612  613  614  615  616  617  618  619  620  621  622  623  624  625  626  627  628  629  630  631  S    F    P    A    A    K    P    K    E    P    T    M    V    A    P    R    A    R    K    E    P    K    E    I    T    TCC  TTC  CCT  GCA  GCC  AAG  CCC  AAG  GAG  CCC  ACC  ATG  GTG  GCC  CCC  CGG  GCC  AGG  AAG  GAG  CCC  AAG  GAG  ATC  ACT  632  633  634  635  636  637  638  639  640  641  642  643  644  645  646  647  648  649  650  651  652  653  654  655  656  F    L    D    A    K    K    S    L    N    L    N    I    F    L    K    Q    F    K    C    S    N    E    E    V    A    TTC  CTC  GAT  GCC  AAG  AAG  AGC  CTG  AAC  CTC  AAC  ATC  TTC  CTG  AAG  CAA  TTT  AAG  TGC  TCC  AAC  GAG  GAG  GTC  GCT  657  658  659  660  661  662  663  664  665  666  667  668  669  670  671  672  673  674  675  676  677  678  679  680  681  A    M    I    R    A    G    D    T    T    K    F    D    V    E    V    L    K    Q    L    L    K    L    L    P    E    GCT  ATG  ATC  CGG  GCT  GGA  GAT  ACC  ACC  AAG  TTT  GAT  GTG  GAG  GTT  CTC  AAA  CAA  CTC  CTT  AAG  CTC  CTT  CCC  GAG  682  683  684  685  686  687  688  689  690  691  692  693  694  695  696  697  698  699  700  701  702  703  704  705  706  K    H    E    I    E    N    L    R    A    F    T    E    E    R    A    K    L    A    S    A    D    H    F    Y    L    AAG  CAC  GAG  ATT  GAA  AAC  CTG  CGG  GCA  TTC  ACA  GAG  GAG  CGA  GCC  AAG  CTG  GCC  AGC  GCC  GAC  CAC  TTC  TAC  CTC  707  708  709  710  711  712  713  714  715  716  717  718  719  720  721  722  723  724  725  726  727  728  729  730  731  L    L    L    A    I    P    C    Y    Q    L    R    I    E    C    M    L    L    C    E    G    A    A    A    V    L    CTC  CTG  CTG  GCC  ATT  CCC  TGC  TAC  CAG  CTG  CGA  ATC  GAG  TGC  ATG  CTG  CTG  TGT  GAG  GGC  GCG  GCC  GCC  GTG  CTG  732  733  734  735  736  737  738  739  740  741  742  743  744  745  746  747  748  749  750  751  752  753  754  755  756  D    M    V    R    P    K    A    Q    L    V    L    A    A    C    E    S    L    L    T    S    R    Q    L    P    I    GAC  ATG  GTG  CGG  CCC  AAG  GCC  CAG  CTG  GTG  CTG  GCT  GCC  TGC  GAA  AGC  CTG  CTC  ACC  AGC  CGC  CAG  CTG  CCC  ATC  757  758  759  760  761  762  763  764  765  766  767  768  769  770  771  772  773  774  775  776  777  778  779  780  781  F    C    Q    L    I    L    R    I    G    N    F    L    N    Y    G    S    H    T    G    D    A    D    G    F    K    TTC  TGC  CAG  CTG  ATC  CTG  AGA  ATT  GGG  AAC  TTC  CTC  AAC  TAC  GGC  AGC  CAC  ACC  GGT  GAC  GCC  GAC  GGC  TTC  AAG  782  783  784  785  786  787  788  789  790  791  792  793  794  795  796  797  798  799  800  801  802  803  804  805  806  I    S    T    L    L    K    L    T    E    T    K    S    Q    Q    N    R    V    T    L    L    H    H    V    L    E    ATC  AGC  ACA  TTG  CTG  AAG  CTC  ACG  GAG  ACC  AAG  TCC  CAG  CAG  AAC  CGC  GTG  ACG  CTG  CTG  CAC  CAC  GTG  CTG  GAG  807  808  809  810  811  812  813  814  815  816  817  818  819  820  821  822  823  824  825  826  827  828  829  830  831  E    A    E    K    S    H    P    D    L    L    Q    L    P    R    D    L    E    Q    P    S    Q    A    A    G    I    GAA  GCG  GAA  AAG  AGC  CAC  CCC  GAC  CTC  CTG  CAG  CTG  CCC  CGG  GAC  CTG  GAA  CAG  CCC  TCG  CAA  GCA  GCA  GGG  ATC  832  833  834  835  836  837  838  839  840  841  842  843  844  845  846  847  848  849  850  851  852  853  854  855  856  N    L    E    I    I    R    S    E    A    S    S    N    L    K    K    L    L    E    T    E    R    K    V    S    A    AAC  CTG  GAG  ATC  ATC  CGC  TCA  GAG  GCC  AGC  TCC  AAC  CTG  AAG  AAG  CTT  CTG  GAG  ACC  GAG  CGG  AAG  GTG  TCT  GCC  857  858  859  860  861  862  863  864  865  866  867  868  869  870  871  872  873  874  875  876  877  878  879  880  881  S    V    A    E    V    Q    E    Q    Y    T    E    R    L    Q    A    S    I    S    A    F    R    A    L    D    E    TCC  GTG  GCC  GAG  GTC  CAG  GAG  CAG  TAC  ACC  GAG  CGC  CTC  CAG  GCC  AGC  ATC  TCG  GCC  TTC  CGG  GCA  CTG  GAC  GAG  882  883  884  885  886  887  888  889  890  891  892  893  894  895  896  897  898  899  900  901  902  903  904  905  906  L    F    E    A    I    E    Q    K    Q    R    E    L    A    D    Y    L    C    E    D    A    Q    Q    L    S    L    CTG  TTT  GAG  GCC  ATC  GAG  CAG  AAG  CAA  CGG  GAG  CTG  GCC  GAC  TAC  CTG  TGT  GAG  GAC  GCC  CAG  CAG  CTG  TCC  CTG  907  908  909  910  911  912  913  914  915  916  917  918  919  920  921  922  923  924  925  926  927  928  929  930  931  E    D    T    F    S    T    M    K    A    F    R    D    L    F    L    R    A    L    K    E    N    K    D    R    K    GAG  GAC  ACG  TTC  AGC  ACC  ATG  AAG  GCT  TTC  CGG  GAC  CTT  TTC  CTC  CGC  GCC  CTG  AAG  GAG  AAC  AAG  GAC  CGG  AAG  932  933  934  935  936  937  938  939  940  941  942  943  944  945  946  947  948  949  950  951  952  953  954  955  956  E    Q    A    A    K    A    E    R    R    K    Q    Q    L    A    E    E    E    A    R    R    P    R    G    E    D    GAG  CAG  GCG  GCG  AAG  GCA  GAG  AGG  AGG  AAG  CAG  CAG  CTG  GCG  GAG  GAG  GAG  GCG  CGG  CGG  CCT  CGG  GGA  GAG  GAC  957  958  959  960  961  962  963  964  965  966  967  968  969  970  971  972  973  974  975  976  977  978  979  980  981  G    K    P    V    R    K    G    P    G    K    Q    E    E    V    C    V    I    D    A    L    L    A    D    I    R    GGG  AAG  CCT  GTC  AGG  AAG  GGG  CCC  GGG  AAG  CAG  GAG  GAG  GTG  TGT  GTC  ATC  GAT  GCC  CTG  CTG  GCT  GAC  ATC  AGG  982  983  984  985  986  987  988  989  990  991  992  993  994  995  996  997  998  999  1000 1001 1002 1003 1004 1005 1006 K    G    F    Q    L    R    K    T    A    R    G    R    G    D    T    D    G    G    S    K    A    A    S    M    D    AAG  GGC  TTC  CAG  CTG  CGG  AAG  ACA  GCC  CGG  GGC  CGC  GGG  GAC  ACC  GAC  GGG  GGC  AGC  AAG  GCA  GCC  TCC  ATG  GAT  1007 1008 1009 1010 1011 1012 1013 1014 1015 1016 1017 1018 1019 1020 1021 1022 1023 1024 1025 1026 1027 1028 1029 1030 1031 P    P    R    A    T    E    P    V    A    T    S    N    P    A    G    D    P    V    G    S    T    R    C    P    A    CCC  CCA  AGA  GCC  ACA  GAG  CCT  GTG  GCC  ACC  AGT  AAC  CCT  GCA  GGA  GAC  CCC  GTG  GGC  AGC  ACG  CGC  TGT  CCC  GCC  1032 1033 1034 1035 1036 1037 1038 1039 1040 1041 1042 1043 1044 1045 1046 1047 1048 1049 1050 1051 1052 1053 1054 1055 1056 S    E    P    G    L    D    A    T    T    A    S    E    S    R    G    W    D    L    V    D    A    V    T    P    G    TCT  GAG  CCC  GGC  CTT  GAT  GCT  ACA  ACA  GCC  AGC  GAG  TCC  CGG  GGC  TGG  GAC  CTT  GTA  GAC  GCC  GTG  ACC  CCC  GGC  1057 1058 1059 1060 1061 1062 1063 1064 1065 1066 1067 1068 1069 1070 1071 1072 1073 1074 1075 1076 1077 1078 1079 1080 1081 P    Q    P    T    L    E    Q    L    E    E    G    G    P    R    P    L    E    R    R    S    S    W    Y    V    D    CCT  CAG  CCC  ACC  CTG  GAG  CAG  TTG  GAG  GAG  GGT  GGT  CCC  CGG  CCC  CTG  GAG  AGG  CGT  TCT  TCC  TGG  TAT  GTG  GAT  1082 1083 1084 1085 1086 1087 1088 1089 1090 1091 1092 1093 1094 1095 1096 1097 1098 1099 1100 1101 1102 1103 1104 1105 1106 A    S    D    V    L    T    T    E    D    P    Q    C    P    Q    P    L    E    G    A    W    P    V    T    L    G    GCC  AGC  GAT  GTC  CTA  ACC  ACT  GAG  GAT  CCC  CAG  TGC  CCC  CAG  CCC  TTG  GAG  GGG  GCC  TGG  CCG  GTG  ACT  CTG  GGA  1107 1108 1109 1110 1111 1112 1113 1114 1115 1116 1117 1118 1119 1120 1121 1122 1123 1124 1125 1126 1127 1128 1129 1130 1131 D    A    Q    A    L    K    P    L    K    F    S    S    N    Q    P    P    A    A    G    S    S    R    Q    D    A    GAT  GCT  CAG  GCC  CTG  AAG  CCC  CTC  AAG  TTC  TCC  AGC  AAC  CAG  CCC  CCT  GCA  GCC  GGA  AGT  TCA  AGG  CAA  GAT  GCC  1132 1133 1134 1135 1136 1137 1138 1139 1140 1141 1142 1143 1144 1145 1146 1147 1148 1149 1150 1151 1152 1153 1154 1155 1156 K    D    P    T    S    L    L    G    V    L    Q    A    E    A    D    S    T    S    E    G    L    E    D    A    V    AAG  GAT  CCC  ACG  TCC  TTG  CTG  GGC  GTC  CTC  CAG  GCC  GAG  GCC  GAC  AGC  ACA  AGT  GAG  GGG  CTG  GAG  GAC  GCT  GTC  1157 1158 1159 1160 1161 1162 1163 1164 1165 1166 1167 1168 1169 1170 1171 1172 1173 1174 1175 1176 1177 1178 1179 1180 1181 H    S    R    G    A    R    P    P    A    A    G    P    G    G    D    E    D    E    D    E    E    D    T    A    P    CAC  AGC  CGT  GGT  GCC  AGA  CCC  CCT  GCA  GCA  GGC  CCA  GGT  GGG  GAT  GAG  GAC  GAG  GAC  GAG  GAG  GAC  ACG  GCC  CCA  1182 1183 1184 1185 1186 1187 1188 1189 1190 1191 1192 1193 1194 1195 1196 1197 1198 1199 1200 1201 1202 1203 1204 1205 1206 E    S    A    L    D    T    S    L    D    K    S    F    S    E    D    A    V    T    D    S    S    G    S    G    T    GAG  TCC  GCA  CTG  GAC  ACA  TCC  CTG  GAC  AAG  TCC  TTC  TCC  GAG  GAT  GCG  GTG  ACC  GAC  TCC  TCG  GGG  TCG  GGC  ACA  1207 1208 1209 1210 1211 1212 1213 1214 1215 1216 1217 1218 1219 1220 1221 1222 1223 1224 1225 1226 1227 1228 1229 1230 1231 L    P    R    A    R    G    R    A    S    K    G    T    G    K    R    R    K    K    R    P    S    R    S    Q    E    CTC  CCC  AGG  GCC  CGG  GGC  CGG  GCC  TCA  AAG  GGG  ACC  GGG  AAG  CGA  AGG  AAG  AAG  CGT  CCC  TCC  AGG  AGC  CAG  GAA  1232 1233 1234 1235 1236 1237 1238 1239 1240 1241 1242 1243 1244 1245 1246 1247 1248 1249 E    V    P    P    D    S    D    D    N    K    T    K    K    L    C    V    I    Q    .   GAG  GTT  CCC  CCT  GAT  TCT  GAT  GAT  AAT  AAA  ACA  AAG  AAA  CTG  TGT  GTG  ATC  CAG  TAAGGTACCCCGGCTGCTAAC  DAAM2-FFC (NP_001188356.1, aa 486-1068)                                                            486  487  488  489  490  491  492  493  494  495  496  497  498  M    G    S    S    H    H    H    H    H    H    G    S    M    K    D    K    L    A    R    E    S    Q    E    L    R    ATG  GGC  AGC  AGC  CAT  CAT  CAT  CAT  CAT  CAC  GGA  TCC  ATG  AAA  GAT  AAG  TTG  GCC  CGT  GAA  AGC  CAA  GAG  CTG  CGC  499  500  501  502  503  504  505  506  507  508  509  510  511  512  513  514  515  516  517  518  519  520  521  522  523  Q    A    R    G    Q    V    A    E    L    V    A    Q    L    S    E    L    S    T    G    P    V    S    S    P    P    CAG  GCT  CGT  GGA  CAG  GTC  GCA  GAG  TTA  GTT  GCT  CAA  TTA  TCA  GAG  CTT  TCT  ACA  GGC  CCA  GTT  TCG  TCC  CCG  CCG  524  525  526  527  528  529  530  531  532  533  534  535  536  537  538  539  540  541  542  543  544  545  546  547  548  P    P    G    G    P    L    T    L    S    S    S    M    T    T    N    D    L    P    P    P    P    P    P    L    P    CCA  CCA  GGA  GGG  CCC  CTG  ACC  TTA  TCC  AGC  TCG  ATG  ACC  ACC  AAC  GAT  CTG  CCG  CCC  CCT  CCG  CCG  CCC  TTA  CCT  549  550  551  552  553  554  555  556  557  558  559  560  561  562  563  564  565  566  567  568  569  570  571  572  573  F    A    C    C    P    P    P    P    P    P    P    L    P    P    G    G    P    P    T    P    P    G    A    P    P    TTT  GCG  TGT  TGT  CCG  CCC  CCG  CCA  CCT  CCA  CCG  CTT  CCC  CCA  GGC  GGA  CCG  CCA  ACG  CCA  CCG  GGA  GCA  CCA  CCT  574  575  576  577  578  579  580  581  582  583  584  585  586  587  588  589  590  591  592  593  594  595  596  597  598  C    L    G    M    G    L    P    L    P    Q    D    P    Y    P    S    S    D    V    P    L    R    K    K    R    V    TGC  CTG  GGC  ATG  GGG  TTA  CCA  TTA  CCT  CAA  GAT  CCT  TAC  CCC  AGC  AGC  GAT  GTA  CCA  TTA  CGC  AAA  AAA  CGT  GTC  599  600  601  602  603  604  605  606  607  608  609  610  611  612  613  614  615  616  617  618  619  620  621  622  623  P    Q    P    S    H    P    L    K    S    F    N    W    V    K    L    N    E    E    R    V    P    G    T    V    W    CCG  CAA  CCA  TCT  CAT  CCC  TTA  AAA  TCG  TTC  AAT  TGG  GTC  AAA  CTG  AAC  GAA  GAA  CGC  GTA  CCT  GGT  ACT  GTG  TGG  624  625  626  627  628  629  630  631  632  633  634  635  636  637  638  639  640  641  642  643  644  645  646  647  648  N    E    I    D    D    M    Q    V    F    R    I    L    D    L    E    D    F    E    K    M    F    S    A    Y    Q    AAT  GAG  ATC  GAT  GAT  ATG  CAA  GTT  TTT  CGT  ATC  CTG  GAC  CTG  GAG  GAC  TTC  GAG  AAG  ATG  TTT  AGT  GCC  TAC  CAG  649  650  651  652  653  654  655  656  657  658  659  660  661  662  663  664  665  666  667  668  669  670  671  672  673  R    H    Q    K    E    L    G    S    T    E    D    I    Y    L    A    S    R    K    V    K    E    L    S    V    I    CGT  CAT  CAG  AAG  GAA  CTT  GGC  TCA  ACG  GAG  GAC  ATT  TAT  CTG  GCG  TCG  CGT  AAG  GTC  AAA  GAA  CTT  TCG  GTT  ATT  674  675  676  677  678  679  680  681  682  683  684  685  686  687  688  689  690  691  692  693  694  695  696  697  698  D    G    R    R    A    Q    N    C    I    I    L    L    S    K    L    K    L    S    N    E    E    I    R    Q    A    GAT  GGA  CGT  CGT  GCG  CAG  AAC  TGC  ATT  ATT  TTG  CTG  TCA  AAA  CTT  AAG  TTG  TCG  AAT  GAG  GAA  ATC  CGC  CAA  GCG  699  700  701  702  703  704  705  706  707  708  709  710  711  712  713  714  715  716  717  718  719  720  721  722  723  I    L    K    M    D    E    Q    E    D    L    A    K    D    M    L    E    Q    L    L    K    F    I    P    E    K    ATC  CTG  AAG  ATG  GAC  GAG  CAA  GAA  GAC  TTG  GCG  AAA  GAT  ATG  TTA  GAG  CAA  CTG  CTT  AAG  TTT  ATT  CCA  GAG  AAG  724  725  726  727  728  729  730  731  732  733  734  735  736  737  738  739  740  741  742  743  744  745  746  747  748  S    D    I    D    L    L    E    E    H    K    H    E    I    E    R    M    A    R    A    D    R    F    L    Y    E    AGT  GAC  ATC  GAT  TTG  TTG  GAA  GAG  CAC  AAG  CAT  GAA  ATT  GAA  CGC  ATG  GCG  CGT  GCC  GAC  CGC  TTT  CTG  TAT  GAA  749  750  751  752  753  754  755  756  757  758  759  760  761  762  763  764  765  766  767  768  769  770  771  772  773  M    S    R    I    D    H    Y    Q    Q    R    L    Q    A    L    F    F    K    K    K    F    Q    E    R    L    A    ATG  TCT  CGC  ATT  GAT  CAT  TAT  CAG  CAA  CGC  TTA  CAG  GCA  CTG  TTT  TTC  AAA  AAG  AAA  TTT  CAG  GAG  CGT  TTG  GCA  774  775  776  777  778  779  780  781  782  783  784  785  786  787  788  789  790  791  792  793  794  795  796  797  798  E    A    K    P    K    V    E    A    I    L    L    A    S    R    E    L    V    R    S    K    R    L    R    Q    M    GAA  GCA  AAA  CCC  AAA  GTT  GAA  GCA  ATT  TTA  CTT  GCT  TCT  CGT  GAA  TTG  GTA  CGT  AGC  AAG  CGT  TTA  CGC  CAG  ATG  799  800  801  802  803  804  805  806  807  808  809  810  811  812  813  814  815  816  817  818  819  820  821  822  823  L    E    V    I    L    A    I    G    N    F    M    N    K    G    Q    R    G    G    A    Y    G    F    R    V    A    CTG  GAG  GTC  ATC  TTG  GCA  ATC  GGA  AAT  TTT  ATG  AAC  AAG  GGA  CAA  CGC  GGT  GGT  GCA  TAT  GGC  TTC  CGT  GTG  GCA  824  825  826  827  828  829  830  831  832  833  834  835  836  837  838  839  840  841  842  843  844  845  846  847  848  S    L    N    K    I    A    D    T    K    S    S    I    D    R    N    I    S    L    L    H    Y    L    I    M    I    TCA  CTG  AAT  AAG  ATC  GCA  GAT  ACC  AAG  TCC  TCT  ATC  GAC  CGT  AAT  ATC  AGC  CTG  CTT  CAC  TAT  TTA  ATC  ATG  ATC  849  850  851  852  853  854  855  856  857  858  859  860  861  862  863  864  865  866  867  868  869  870  871  872  873  L    E    K    H    F    P    D    I    L    N    M    P    S    E    L    Q    H    L    P    E    A    A    K    V    N    TTA  GAG  AAG  CAT  TTC  CCT  GAC  ATC  CTG  AAC  ATG  CCG  TCT  GAA  TTA  CAG  CAT  TTG  CCA  GAA  GCC  GCA  AAG  GTA  AAC  874  875  876  877  878  879  880  881  882  883  884  885  886  887  888  889  890  891  892  893  894  895  896  897  898  L    A    E    L    E    K    E    V    G    N    L    R    R    G    L    R    A    V    E    V    E    L    E    Y    Q    CTG  GCA  GAA  CTG  GAA  AAA  GAA  GTT  GGT  AAC  CTT  CGC  CGT  GGT  TTA  CGC  GCT  GTA  GAA  GTT  GAG  TTA  GAA  TAT  CAG  899  900  901  902  903  904  905  906  907  908  909  910  911  912  913  914  915  916  917  918  919  920  921  922  923  R    R    Q    V    R    E    P    S    D    K    F    V    P    V    M    S    D    F    I    T    V    S    S    F    S    CGC  CGT  CAG  GTT  CGC  GAG  CCG  TCC  GAC  AAG  TTC  GTC  CCT  GTA  ATG  TCC  GAC  TTT  ATC  ACG  GTA  TCG  TCG  TTT  AGC  924  925  926  927  928  929  930  931  932  933  934  935  936  937  938  939  940  941  942  943  944  945  946  947  948  F    S    E    L    E    D    Q    L    N    E    A    R    D    K    F    A    K    A    L    M    H    F    G    E    H    TTC  TCT  GAA  TTA  GAG  GAC  CAA  TTG  AAT  GAA  GCC  CGC  GAT  AAA  TTT  GCA  AAA  GCG  CTT  ATG  CAT  TTT  GGC  GAG  CAC  949  950  951  952  953  954  955  956  957  958  959  960  961  962  963  964  965  966  967  968  969  970  971  972  973  D    S    K    M    Q    P    D    E    F    F    G    I    F    D    T    F    L    Q    A    F    S    E    A    R    Q    GAC  TCC  AAA  ATG  CAG  CCC  GAT  GAA  TTT  TTC  GGC  ATC  TTC  GAT  ACA  TTT  CTT  CAA  GCA  TTC  TCC  GAG  GCC  CGT  CAA  974  975  976  977  978  979  980  981  982  983  984  985  986  987  988  989  990  991  992  993  994  995  996  997  998  D    L    E    A    M    R    R    R    K    E    E    E    E    R    R    A    R    M    E    A    M    L    K    E    Q    GAC  CTG  GAA  GCA  ATG  CGC  CGT  CGT  AAG  GAG  GAG  GAA  GAG  CGC  CGC  GCT  CGC  ATG  GAA  GCG  ATG  CTT  AAA  GAG  CAG  999  1000 1001 1002 1003 1004 1005 1006 1007 1008 1009 1010 1011 1012 1013 1014 1015 1016 1017 1018 1019 1020 1021 1022 1023 R    E    R    E    R    W    Q    R    Q    R    K    V    L    A    A    G    S    S    L    E    E    G    G    E    F    CGT  GAG  CGC  GAA  CGT  TGG  CAA  CGT  CAG  CGT  AAG  GTA  TTA  GCA  GCA  GGT  TCG  TCT  TTA  GAA  GAG  GGC  GGT  GAA  TTT  1024 1025 1026 1027 1028 1029 1030 1031 1032 1033 1034 1035 1036 1037 1038 1039 1040 1041 1042 1043 1044 1045 1046 1047 1048 D    D    L    V    S    A    L    R    S    G    E    V    F    D    K    D    L    C    K    L    K    R    S    R    K    GAC  GAC  CTT  GTT  AGC  GCA  CTG  CGT  TCG  GGG  GAA  GTT  TTT  GAT  AAG  GAT  CTT  TGT  AAA  TTA  AAG  CGT  TCG  CGT  AAG  1049 1050 1051 1052 1053 1054 1055 1056 1057 1058 1059 1060 1061 1062 1063 1064 1065 1066 1067 1068 R    S    G    S    Q    A    L    E    V    T    R    E    R    A    I    N    R    L    N    Y    .    CGT  AGC  GGC  TCC  CAA  GCC  TTG  GAA  GTG  ACA  CGC  GAG  CGC  GCG  ATT  AAT  CGC  TTA  AAT  TAT  TGA  GGTACCCCGGCTGCTAAC                                                                                                                     
