# Supplementary material for: Alterations to the broad-spectrum formin inhibitor SMIFH2 modulate potency but not specificity
Source: Sci Rep. 2022 Aug 8;12:13520. doi: 10.1038/s41598-022-17685-z (PMC9360399; doi:10.1038/s41598-022-17685-z)
Supplement: Supplementary file 5 — Supplementary Information 5. [file 41598_2022_17685_MOESM5_ESM.pdf]

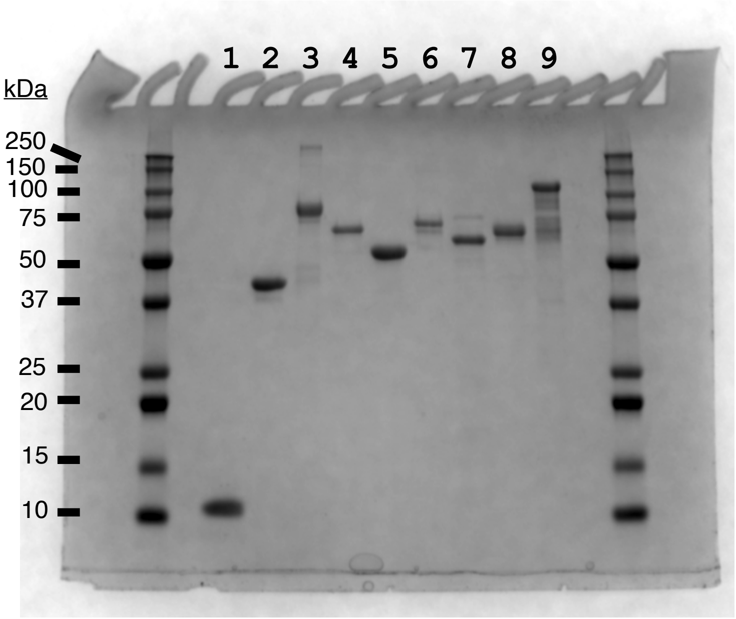

1	profilin
2	actin
3	DIAPH1-FFC
4	DIAPH2-FFC
5	Delphilin-FFC
6	DAAM2-FFC
7	FMN2-FFC
8	FMNL3-FFC
9	INF2-FFC

Figure S7. Uncropped gel image of purified proteins (related to Figure 1C). SDS-PAGE analysis of purified proteins with Bio-rad AnyKD gel and Coomassie staining. Molecular weight markers are on each side of the gel and labeled on the left side.
